# Medical and Philosophical Intelligence

**Published:** 1813-01

**Authors:** 


### MEDICAL AND PHILOSOPHICAL INTELLIGENCE.

### *REPORT of the COMMITTEE of APOTHECARIES.*

THE Committee of Apothecaries witness, with peculiar satisfaction, the zeal and alacrity with which the ninth Resolution has already been acted on in Kent, Surrey, Middlesex, Essex, Sussex, Herts, Berks, and the adjacent counties, whence many respectable practitioners came to the general meetings in London; and they hope that that the circulation of the Report and Resolutions will extend a similar spirit to the more remote counties. These excellent intentions will be best promoted by some one or more individuals being deputed to correspond with the London Committee, to collect contributions, and to transmit them, with the names and residence of all practitioners within the district, and any other useful information. Unless gentlemen voluntarily comply with these suggestions, the efforts of the profession cannot be, as they ought, so vigorous and simultaneous as to ensure success. Communications of their proceedings should likewise be promptly made to the more distant parts of the county.

At the general meeting it was liberally voted, that the subscription should be *two,* instead of *one,* guinea; which many gentleman *erroneously suppose excludes the smaller sum.* The Committee conceive that numbers as well as property are essential to a successful event. They, therefore, take the liberty to declare, that they will thankfully receive any donations which may be offered, and that the names of subscribers will be regularly published, without specifying the sum subscribed.

The Committee beg leave to hint to their brethren, that, when their proceedings are sufficiently matured to appear in Parliament, it will be necessary that every individual exert his influence with his parliamentary representatives for an impartial consideration of their appeal, and for their steady support, if approved.

They have to apologise to those gentlemen who have not yet received the Report and Resolutions; and to those who may have been put to the expense of duplicates. These errors are imputable alone to the want of a correct register of names and residences of country practitioners.

Having been unfortunately deprived, from indisposition, of the zealous services of Mr. Powell, the Committee have elected Mr. W. Tilleard Ward, of Holles Street, Cavendish Square, secretary; to whom they request all future communications may be directed.

*December* 22, 1812. W. WARD, Secretary.

The investigation which now occupies the attention and labors of the *Committee of Apothecaries,* is of sufficient importance to the public in general, and to that class of practitioners designated by the appellation SURGEON-APOTHECARY in particular, to justify us for again noticing it in the same Number of our Journal. If we have thought it a duty owed by us to the medical public, and to the deeply-serious cause in which the *Committee of Apothecaries* is now engaged, to make the projected REFORM a prominent feature in our half-yearly *Report;* we feel an impulse equally strong, if we have been misinformed of facts, have erred in opinion, or been too free in observation, to admit our mistakes: and, we hope, with that fairness and candor candor of character which we have felt no common solicitude to impress on the public belief, to explain fully and freely "the head and front of our offending."

Our Report consists of an historical relation, illustrated by observations, and, perhaps, rendered somewhat too picquant by opinions hazarded in a language honest at least, if not courtly. On all these points, subsequent information has demanded some explanatory remarks, essential to a cause we value above all others—the cause of TRUTH.

At the foot of page 5 of our Report, it is lamented that the proposal to admit country practitioners into the committee was rejected. This we understood to be the fact, certainly, to the meeting of the 20th of November; and it will appear in the note, page 7, that we then came to the knowledge that the Committee had acceded to that proposal. It is right to state, however, and we do it on information from highly-respectable members of the Committee, that the proposal for associating country practitioners with the London Committee, was adopted, at the meeting on the 6th of November, with a promptness that affords evidence of the honorable views of the London Committee.

It has been suggested that there are two passages in the Report that may lead to an injurious conclusion, or a wrong apprehension. The first objectionable passage is in page 4, running in the following terms: "*To make a law to protect the rights and privileges of persons incapable of performing the functions of a station into which they have obtruded, is an absurdity.*" This passage certainly involves a great and important difficulty. All retrospective effect will be, perhaps, impracticable; and it is to be regretted that it must be so. For the passage as it stands, the Committee is not, however, in any wise amenable. It is the opinion of the writer of the Report, written without the knowledge of the Committee; and, right or wrong, the Committee is entirely and fully exonerated from all concurrence or participation in it. We may go further, and assure our readers, on the best authority, that the Committee does not intend any of its proceedings to have an *ex post facto* effect, either directly or indirectly, understood or implied. The second passage, objectionable on account of the ambiguity that occurs in the note at page 7, will be readily explained. It was never meant to convey to the public the idea, that the whole of the passage beginning with "Good God," was uttered by the worthy member of the Committee alluded to. Good or bad, true or false, that gentleman is entitled to no more of it than the exclamation "*Good God!*" But there is still another passage, the construction of which we regret, because it may be painful to an individual. To give point to an observation, we set in opposition the county of York and the village of Putney. It was by accident, however, that this village was selected. Hampstead, Plaistow, or any of the others, would have answered; for it never was the design of the reporter to aim a sarcasm at any gentleman who resides at Putney.

The principle of the *Report* we will say was designed to do service to the cause. If we have mistaken the means, or have wanted intelligence ligence in their employment, we take shame to ourselves. But we assert again, and, if we fail in putting the assertion with appropriate force, we beg to have it explicitly known, that we fully believe the Committee to be honorably and "*honestly laboring for the melioration of the condition of the* SURGEON-APOTHECARY, *for the benefit of science, and for the good of society.*" Instead of intending to raise suspicion, we have endeavored to remove it. Instead of retarding the premeditated *Reform*, we have pointed out, according to the best of our judgment, the most effectual means for its accomplishment. Upon this we rest. The fair fame of the Committee we have not impeached, because we did not see that it was impeachable. But men will differ in opinion. We claim the universal privilege. When it is shewn to us where we have erred, we submit to correction: and our pages will be open to liberal and candid animadversions from those who do not think as we do on this important subject.

---

*Philosophical Society of London.*—The attention of the society during the last month has been directed to the subject of Hearing, by a Lecture on the Anatomy and Physiology of the Organ of Hearing in Man and other Animals, by Mr. T. J. Pettigrew.

The lecture commenced by observing the difficulty of treating anatomical and physiological subjects in a sufficiently popular manner to interest a general audience; and by professing an endeavor to divert it of technical phrases as much as possible. The lecturer was aware that, in many instances, in so complicated an organ as the ear, it was utterly impossible to avoid the use of technical terms, but those he endeavored to explain; for the difficulty is exceedingly great when we consider that they are derived, not only from the Hebrew, Arabic, Greek, Latin, French, Italian, Spanish, German, and English, languages; but even from the Indian, African, and Mexican, as Mr. John Mason Good has shewn in a paper on Medical Technology, in the first part of a new series of the Transactions of the Medical Society of London. Notwithstanding the many difficulties which thus presented themselves in the demonstration, Mr. P., conscious of the utility of publicly disseminating so important a branch of knowledge, was emboldened to proceed to his subject with all its imperfections on its head.

The ear he divided, in common with anatomists, into external and internal: the former comprehending the lobulus, pinna, and meatus auditorius externus; the latter the tympanum, labyrinth, and meatus internus. These several parts, together with the eustachian tube and the auditory nerves, were perspicuously demonstrated by diagrams and preparations, and rendered intelligible to the audience.

After this, Mr. P. proceeded to consider sound, and its effects, and to apply them to the phenomena of hearing. He traced the sonorous undulations passing through the air, collected into the concha, and conveyed along the canalis auditorius to the membrana tympani, where he noticed the discovery of a radiated muscle by Sir Everard Home, first in the elephant, and afterwards in man, by which the membrane is brought into a state capable of acting, and of giving those different degrees of tension which empower it to correspond with the variety of external tremors: when the membrane is relaxed, the radiated muscle cannot act with any effect, and external tremors make less accurate impressions.* From the membrane he traced the progress of sound along the opercula auditus, and considered the muscles belonging to them. From the last of the bones (the stapes) he traced it through the fenestre ovalis into the vestibulum and semicircular canals, and treated of the membranes lining those cavities, the fluid contained in them, and the nerves expanded upon the membranes. The cochlea, spiral laminæ, &c. then met with consideration, which parts, the lecturer conceived, were subservient to the diversity of sounds; and concluded the human demonstration by some appropriate observations on the eustachiean tube.

The concluding part of the lecture consisted of comparative anatomical observations.

All vertebral animals, i. e. red-blooded animals, have an organ of hearing: it is likewise found in many insects, and in some of the mollusca.

An abstract of this part we shall thus arrange according to the classes adopted by Linnæus:

*Mammalia*—Form of the ear various. In the porcupine it resembles the human. The cartilage composing the pinna more delicate than in man. The meatus auditorius externus, in animals whose nature leads them to dive or burrow in the ground, furnished with a valve which can be opened and shut at pleasure. Ex. Water-shrew and mole.

Tympanum in all, membrane belonging to it, and an eustachian tube entering it, passing from the fauces, except in the cetacea, where it opens into the blowing hole.

Mastoid cells, or some structure approaching to it, inmost; cellular in the pig, divided by bony septa—a mere cavity in the dog, cat, and others.

Opercula auditus, generally four in number; ornithorynchus paradoxus, only two.

Labyrinth in all quadrupeds. Cochlea sometimes 3½ turns; separate in the whale; semicircular canals small.

*Aves*—No external ear; feathers peculiarly disposed around the meatus, which is valvular. Tympana communicates with each other by cellular structure;† membrane convex externally. Operculum auditus single, connecting the membrane with the vestibulum.

Labyrinth. No cochlea—hollow bony process of similar conformation; semicircular canals projecting from the skull.

*Amphibia*—Crocodile the only one having an external meatus.‡ Many possess tympanum, eustachian tube, and semicircular canals, as the turtle, in whom the membrane is cartilaginous.

---

* Vide Croonian Lecture, Phil. Trans. part 1st, 1800.

† The elephant has a similar structure.

‡ Of which the lecturer produced a beautiful preparation.

A single

A single ossiculum. Frogs two ossicula. Concretions in the vestibulum.

*Pisces*—Internal ear grows proportionately to the size of the animal. Semicircular canals very conspicuous. Concretions of a very brittle nature in the vestibulum.

*Insecta*—Power of hearing doubted by many anatomists, but proved by Minasi, Scarpa, Comparetti, and others. Vestibulum discernible in the Cancer genus. Same nerves as supply the antennæ.

*Vermes*—Only distinct instance the sepia officinalis; vestibulum with concretions.

The lecturer concluded by observing, that, throughout the animal kingdom, wherever there appeared an organ of hearing, a vestibulum containing a fluid, and surrounded by membranes, upon which the nerves are distributed, was conspicuous: this, therefore, he conceived to be the most important part for executing the functions of hearing. In the cuttle-fish it is a mere capsule; ascending in the animal kingdom, other parts are super-added, until it reaches to man, in whom the organ is most complicated and most perfect, as he can not only distinguish simple vocal modulations and articulations, but also the different qualities with relation to one sound, and that too with wonderful accuracy.*

After the lecture, the learned president, Dr. Lettsom, adjourned the society until the 7th of January, with a very neat and appropriate speech.

---

*Deaths by Small-pox weekly, from the London bills.*

| | | | |
|---|---|---|---|
| Nov. 10, | 40 | Nov. 24 | 52 |
| 17, | 42 | Dec. 1 | 45 |

A correspondent observes, in the last Journal, a notice of two cases of small-pox subsequent to vaccination, stated in general terms to be "*one in London and one at Hackney.*"

Now it is submitted that this mode of communicating such facts is likely to create alarm, and injure the cause of vaccination, without the least benefit to science. The suppression of the party's name, and the particular circumstances of the case, obviously shut out the possibility of a proper inquiry; and, if such a practice be permitted, it will be in the power of any adversary of vaccination, however contemptible, to report as many failures as he may think convenient for his purposes. It is, therefore, hoped that the editors will, in their next number, state at length the particulars of the two cases adverted to.† C. M.

*Dec.* 12, 1812.

---

The following comparative statement, taken from an exact register of *three thousand one hundred and ninety-four* patients relieved by the

---

* See Cuvier Leçons d'Anatomie comparée, tome ii.

† Vide p. 87 of the present Number.

New Rupture Society, to December 17, 1812, will shew the proportion and species of herniary cases occurring in each sex:

| | | | *Males.* | *Females.* |
|---|---|---|---|---|
| 760 Double Ruptures | | In both groins | 715 | 14 |
| | | In both thighs | 3 | 28 |
| 2152 Single Ruptures | Inguinal | On the right side | 1207 | 38 |
| | | On the left side | 691 | 25 |
| | Femoral | On the right side | 15 | 101 |
| | | On the left side | 7 | 68 |
| | | Navel ruptures | 21 | 141 |
| | | Ventral herniæ | 3 | 9 |
| | | Prolapses of the anus | 13 | 14 |
| | | Prolapses of the uterus | 0 | 80 |
| | | Prolapses of the urinary bladder | 0 | 1 |
| | | | 2675 | 519 |
| | | | | 2675 |
| | | Total number of cases | | 3194 |

It is difficult to ascertain at what age persons are most liable to become ruptured; probably, however, during the middle period of life: but, not fewer than one hundred of the above patients (nearly all males) were ruptured prior to their birth; and, above the proportion of a fifth of them had double herniæ.

Of the fourteen females afflicted with prolapses of the anus, twelve had also uterine prolapses, one had a navel rupture, and another a prolapse of the urinary bladder. Several of the patients having ruptures in the navel, had at the same time inguinal or femoral herniæ; and one woman had also a prolapsus ani. A person who labored under a ventral rupture, had likewise two inguinal herniæ. One man had prolapsus ani and inguinal rupture. Another male subject had one inguinal and two femoral ruptures, produced by sudden bodily exertion. Several females, having prolapses of the uterus, had also herniæ on one or both sides. And, in many men, hydrocele was combined with their ruptures; all which cases were cured, or relieved, by appropriate surgical operations.

---

Mr. John Cormack, of Forry, in the county of Caithness, has this season reared a few seeds of salmon oats. Five grains were planted at the distance of six inches from each other; the product of which were 150 stalks, bearing 2,550 grains!

---

Mr. William Bullock is arranging the materials of a splendid work, relative to the most recent discoveries in Natural History, with engravings, colored from original specimens.

---

Mr. Francis Gold, of Bristol, member of the Royal College of Surgeons in London, who, on his return from Egypt, was detained in in France by the arrêt of the 23d of May, 1803, and has lately been liberated at the intercession of Dr. Jenner, is at present occupied on the translation of Bichat's work, "*Sur la vie, et la mort,*"—a treatise containing the principles of the Physiology now prevailing in the French schools.

---

A Third Edition of Dr. Wilson's Treatise on Febrile Diseases, is in the press, and will be published early in the Spring, in 2 vols. octavo.

---

Mr. Stevenson's promised work on Cataract, is in the press.

---

Mr. Charles Fothergill has in the press an Essay on the Philosophy, Study, and Use of Natural History, in which some new and original views relating to the science are developed.

---

*Theatre of Anatomy.*—Mr. John Taunton, F.A.S. member of the Royal College of Surgeons of London, surgeon to the City and Finsbury Dispensaries, City of London Truss Society, &c. will commence his Winter Course of Lectures on Anatomy, Physiology, Pathology, and Surgery, on Saturday, January the 23d, 1813, at eight o'clock in the evening, precisely, and continue them every Tuesday, Thursday, and Saturday, at the same hour, at his house, Greville Street, Hatton Garden.

In this course of Lectures it is proposed to take a comprehensive view of the structure and economy of the living body, and to consider the causes, symptoms, nature, and treatment of surgical diseases, with the mode of performing the different surgical operations; forming a complete course of anatomical and physiological instruction for the medical or surgical student, the artist, the professional or private gentleman.

An ample field for professional edification will be afforded by the opportunity which pupils may have of attending the clinical and other practice of both the City and Finsbury Dispensaries.

---

*Theatre of Anatomy, Blenheim-street, Great Marlborough-street.*—The spring course of Lectures on Anatomy, Physiology, and Surgery, will be commenced on Monday the 25th of January, at two o'clock, by Mr. Brookes.

Anatomical converzationes will be held weekly, when the different subjects treated of will be discussed familiarly, and the student's views forwarded. To these none but pupils can be admitted.

Spacious apartments, thoroughly ventilated, and replete with every convenience, are open all the morning for the purposes of dissecting and injecting, where Mr. Brookes attends to direct the students, and demonstrate the various parts as they appear on dissection.

The inconveniences usually attending anatomical investigations, are counteracted by an antiseptic process.

*London*

*London Hospital.*—Dr. Buxton will commence his spring course of Lectures on the Practice of Medicine, about the 20th of January, 1813.

---

Dr. Squire will, on Thursday the 7th of January, commence a course of Lectures on the Theory and Practice of Midwifery, and the Diseases of Women and Children, at his house, Ely-place, Holborn.

---

Dr. Merriman, Physician-Accoucheur to the Middlesex Hospital, and Westminster General Dispensary, will re-commence his course of Lectures on Midwifery and the Diseases of Women and Children, on Monday, January 4th, at the *Middlesex Hospital.*

---

Mr. Thomas Shute will commence his Spring course of Lectures on Anatomy, Physiology, and the Principles of, and Operations in, Surgery, on Saturday the 9th of January, at eight o'clock in the morning, at the *Anatomical Theatre,* Lower College-street, Bristol.

---

Dr. Adams's Lectures on the Institutes and Practice of Medicine, will commence about the middle of January, at his house in Hatton Garden.

---

Dr. Clutterbuck will begin his Spring course of Lectures on the Theory and Practice of Physic, Materia Medica, and Chemistry, on Monday, January 18th, at ten o'clock in the morning, at his house, No. 1, in the Crescent, New Bridge-street.

---

Dr. Ramsbotham will commence his next course of Lectures on the Science and Practice of Midwifery, on Monday, January 18th, 1813, at half after five in the evening, at his house, No. 9, Old Jewry.

---

Dr. Clarke and Mr. Clarke will commence their Spring course of Lectures on Midwifery, and the Diseases of Women and Children, on Monday, January 25th. The lectures are read every day at the house of Mr. Clarke, No. 10, Upper John-street, Golden-square, from a quarter past ten o'clock in the morning, to a quarter past eleven, for the convenience of students attending the hospital.

METEO-

